# Transcriptome datasets of macrophages infected with different strains of *Leptospira* spp

**DOI:** 10.1016/j.dib.2017.12.042

**Published:** 2017-12-21

**Authors:** Erivelto Corrêa de Araújo Junior, Leandro Encarnação Garcia, Larissa Martins Melo, Jaqueline Poleto Bragato, Valéria Marçal Félix de Lima, Juliana Regina Peiró, Flavia Lombardi Lopes, Márcia Marinho

**Affiliations:** a Department of Support, Production and Animal Health, São Paulo State University (Unesp), School of Veterinary Medicine, Araçatuba, SP, Brazil; b Department of Clinic, Surgery and Animal Reproduction, São Paulo State University (Unesp), School of Veterinary Medicine, Araçatuba, SP, Brazil

**Keywords:** Microarray, *Leptospira*, Macrophage

## Abstract

The datasets reported herein provide information about microarray experiment of macrophage cell line J774A.1 infected with three different strains of *Leptospira spp*. Transcriptomic profiles were generated using Affymetrix® Mouse Gene 2.1 ST Array Strip. Data was normalized and statically process, *p*-value < 0.01, FDR < 0.05 and log2 fold change (± 2). The microarray raw data are available in Gene Expression Omnibus (GEO) under accession number GSE105141.

**Specifications Table**

**Table:** 

Subject area	Biology
More specific subject area	Gene expression
Type of data	Table and Figures
How data was acquired	Microarray Affymetrix® Mouse Gene 2.1 ST Array Strip
Data format	Raw (CEL.)
Experimental factors	cell lines non-infected x cell lines infected with *Leptospira* spp.
Experimental features	Total RNA, with RIN 10, was extracted from whole macrophage cell line infected and control. Each sample contains three biological replicates and each replicates consists of three culture well.
Data source location	Department of Support, Production and Animal Health, São Paulo State University, School of Veterinary Medicine, Araçatuba, SP, Brazil
Data accessibility	Microarray data are available from Gene Expression Omnibus database with GEO accession number GSE105141

**Value of the data**

• This is the first comparison of transcriptomic profile of murine macrophages infected with different *Leptospira* spp varying in species and virulence within strain.
• Data reported will help in understanding the pathogenicity and immune response to leptospirosis which is still poorly understood.
• This data can reveal new insight on modulation and function of genes in immune cells following infection by *Leptospira*, with particular focus on differential host immune response to varying bacterial virulence.

## 1 Data

Total RNA was extracted from murine macrophage cell line J774A.1 infected with virulent, attenuated or saprophyte strains of *Leptospira*, as well as control non-infected cells, 6 h post *in vitro* infection. Affymetrix microarray was performed to obtain transcriptomic profiles of the infected and control groups. Raw data was deposited at NCBI GEO DataSets (GSE105141).

## 2 Experimental design, materials and methods

### 2.1 Cell culture

Murine macrophage cell line J774A.1, provided by the Paul Ehrlich cell bank, Rio de Janeiro, Brazil, was maintained in RPMI-1640 media (Sigma, USA) supplemented with 10% heat-inactivated fetal bovine serum (Gibco, USA), 100 ug/mL streptomycin (Sigma Chemical Co St.Louis, MO), 0.03% L-glutamine solution (Sigma) and 100 UI/mL of penicillin. Cells were incubated at 37 °C, 5% CO2 until formation of a confluent monolayer in 6-well cell culture plates (3 cm/well).

### 2.2 Bacterial culture

Samples of the virulent strain *Leptospira interrogans* sorovar Copenhageni (FIOCRUZ L1-130), attenuated strain *L. interrogans* sorovar Copenhageni M20, and saprophyte strain *L.biflexa* sorovar Patoc (FIOCRUZ -Patoc I) were utilized. Attenuation of sample M20 was performed with successive replications in culture medium. All strains were maintained in Fletcher semi-solid culture medium, and incubated at 30 °C. To restore bacterial virulence (virulent strain), 1 mL of cultured bacteria was inoculated intraperitoneally in Golden Syrian hamsters (*Mesocricetus auratus*) and later recovered from kidneys, whereas the attenuated strain did not undergo intraperitoneal inoculation in hamsters. Virulent leptospires cultures are routinely maintained at the Faculdade de MedicinaVeterinária e Zootecnia, Universidade de São Paulo (USP), São Paulo, Brazil, as previously described [1]. The inoculum was quantified using a Petroff-Hausser chamber.

### 2.3 Infection of macrophages

After the formation of a confluent cell monolayer, cultured cells were washed tree times with sterile phosphate buffer solution (pH 7,2) for removal of antibiotics and non-adherent cells. *L. interrogans* and *L. biflexa* were harvested by centrifugation and the pellet was resuspended in RPMI-1640 media (Sigma), and 100:1 bacteria:cell of the three groups (virulent, attenuated and saprophyte) were added to macrophages and non-infected macrophages were kept as control. All treatments were performed in biological triplicates. Cells (infected and control) were incubated in fresh RPMI medium, without antibiotics, for 6 h at 37 °C, 5% C02. Following this period, RNA extraction was immediately performed as described below.

### 2.4 RNA extraction and quantification

**Fig. 1:**
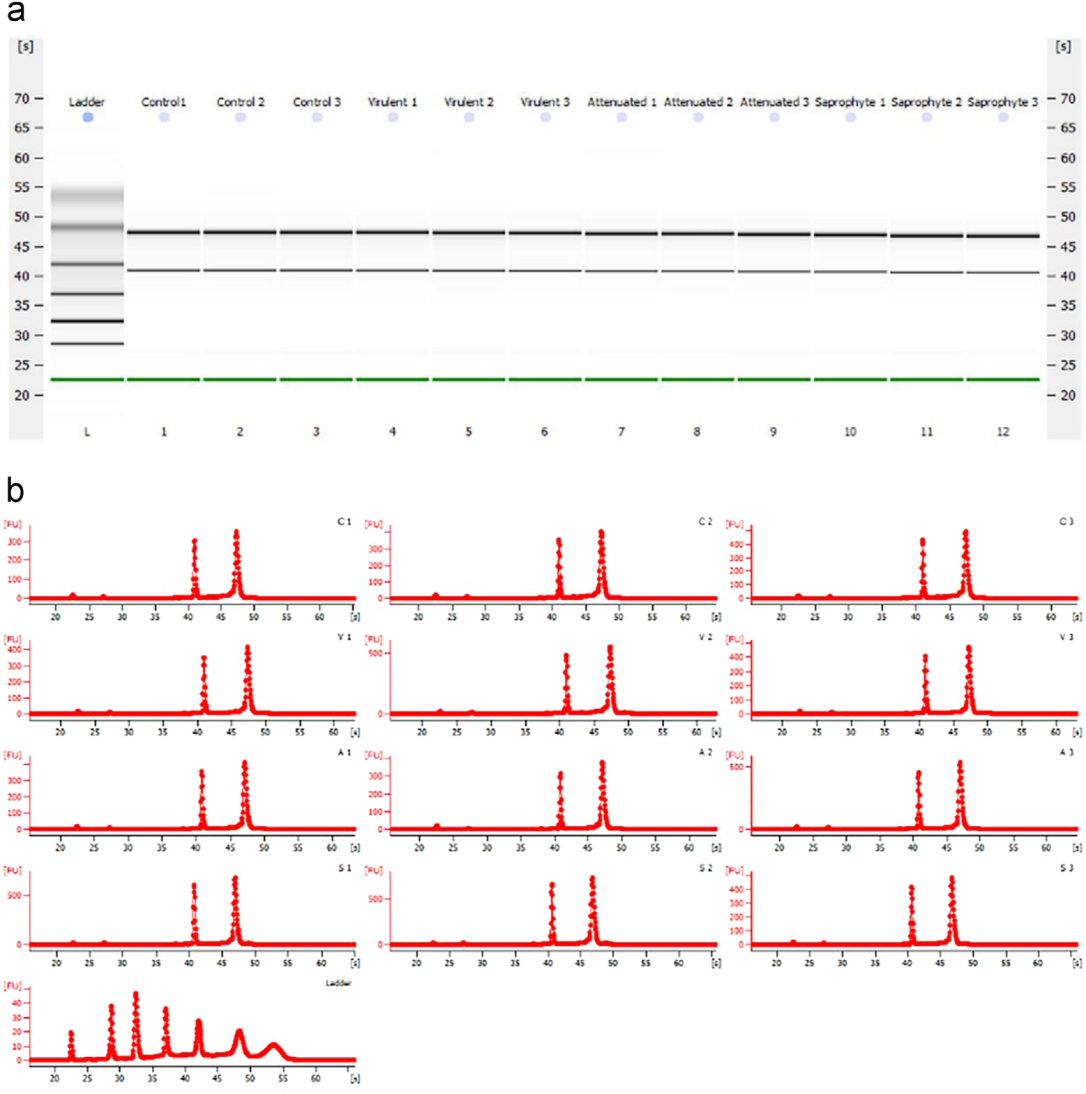
**RNA Quality control.** Total RNA extracted at 6 h post infection and control non-infected was used to analyze concentration and quality. **a)** Bioanalyzer gel image for each sample. **b)** Graphic of all RNA samples used for microarray analysis. The 28S and 18 s distinctive ribosomal RNA bands were observed for all samples.

Total RNA was extracted from macrophages with a RNeasy Mini Kit (Qiagen, USA) according to manufacturer's instructions. RNA samples were immediately stored at −80 °C. Quantification was performed using NanoDrop (ND-2000 spectrophotometer, Thermo Scientific, Wilmington, DE, USA) with OD260/280 ratios between 2,07 and 2,09 and concentration ranging of 1689 to 2818 ng/µl (Table 1). Quality of samples was assessed using capillary electrophoresis (Bioanalyzer 2100 Agilent, Santa Clara, CA, USA) and all samples used for microarray analysis had a RIN of 10 (Fig. 1a–b).

**Table 1:** Spectrophotometric reading of RNA samples.

**Sample**	**Concentration (ng/µL)**	**A260**	**A280**	**260/280**	**260/230**
Control1	2151,3	53,782	25,983	2,07	2,20
Control2	2114,2	52,855	25,484	2,07	2,17
Control3	2497,3	62, 433	30,298	2,06	2,17
Virulent1	2200,2	55,005	26,412	2,08	2,18
Virulent2	2818,2	70,456	33,697	2,09	2,22
Virulent3	1786,1	44,653	21,311	2,10	2,18
Attenuated1	1883,4	47,086	22,535	2,09	2,18
Attenuated2	2397,8	59,946	28,917	2,07	2,21
Attenuated3	2156,5	53,912	25,968	2,08	2,20
Saprófita1	2064,7	51,618	24,734	2,09	2,04
Saprófita2	2742,1	68,553	32,727	2,09	2,12
Saprófita3	1689,1	42,229	20,214	2,09	2,17

### 2.5 Microarray analysis

**Fig. 2:**
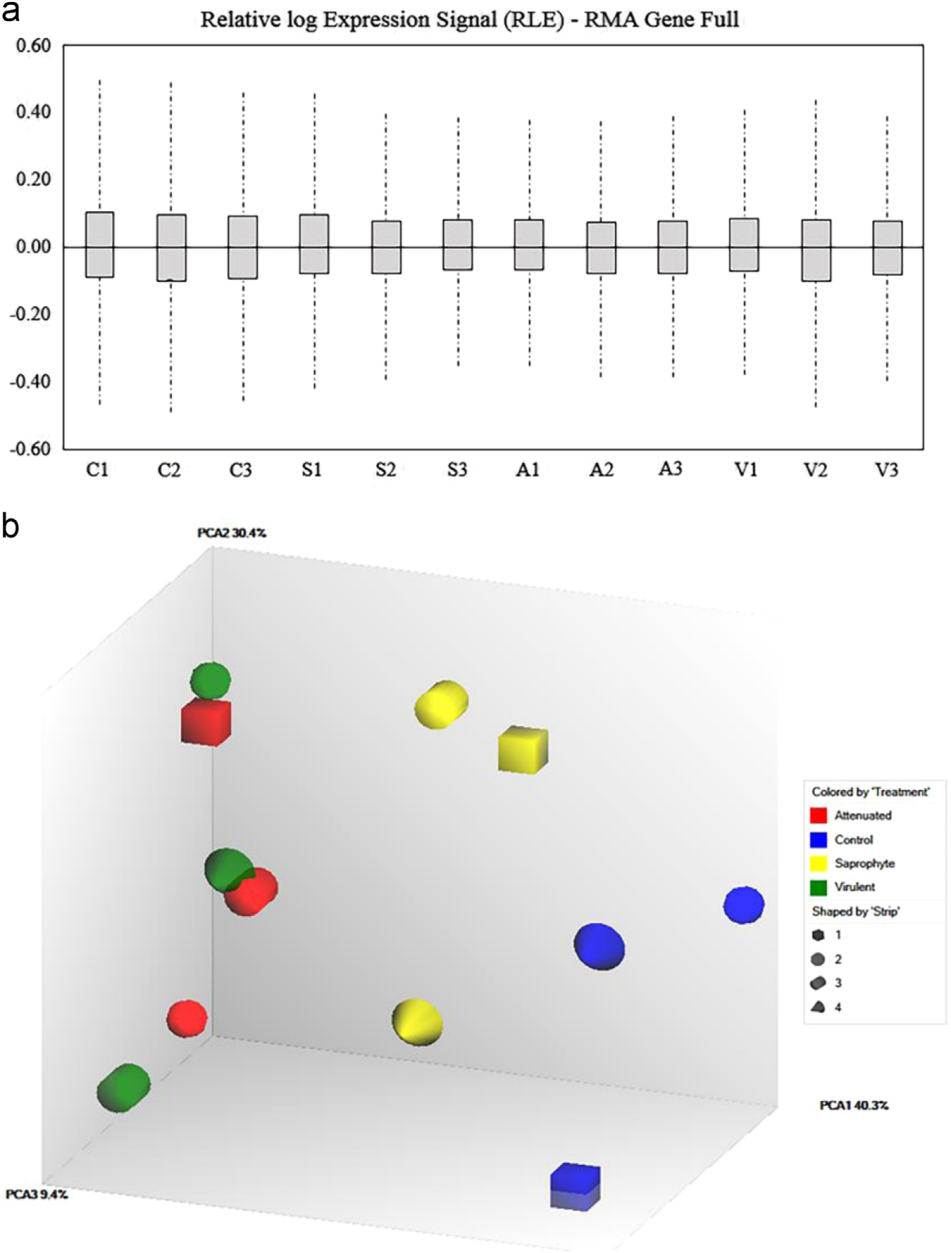
**Signal distribution after normalization. a)** Values of relative log expression signal after RMA-DABG normalization between all samples. (**b)** Principal component Analysis of probe cell intensity for all samples.

A WT PLUS Reagent kit (Affymetrix, Santa Clara, CA, USA) was used to prepare the RNA samples for whole transcriptome expression analysis using the Mouse Genome 2.1 ST Array Strip (Affymetrix), according to the manufacturer´s instructions. Briefly, Poly-A RNA controls (lys, phe, thr and dap), absent in eukaryotic cells, were added to the RNA samples (assay control) prior to generation of cDNA. After the amplification process, final cDNA was purified, quantified, fragmented and labeled for hybridization to strips (all 4 groups were represented in each strip, experiment was repeated three times). Strips remained for 20 h at 48 °C in a hybridization oven. Strips were then washed and stained in the GeneAtlas robotic system (Affymetrix) using the GeneAtlas Hybridization, Wash, and Stain Kit for WT Array Strips (Affymetrix). Finally, strips were scanned using the GeneAtlas Imaging System (Affymetrix) generating the raw cel files. Raw intensity values in the cel files, were background corrected, log2 transformed and then quantile normalized by the software Expression Console (Affymetrix) using the Robust Multi-array Average (RMA) algorithm. Finally, comparison of the relative log expression signal (RLE) by box-plot and Principal Component Analysis (PCA) was performed between samples (Fig. 2a–b). PCA analysis show that infected treatments can be distinguished from controls, with virulent and attenuated groups separating further than the saprophyte bacteria. Next, statistical analysis was performed in the TAC software (Affymetrix) by ANOVA and p-values were corrected for multiple hypothesis testing using the Benjamini-Hochberg procedure (fold change ± 2, FDR-corrected *p* < 0.01).

### 2.6 Identification of differentially expressed genes

**Fig. 3:**
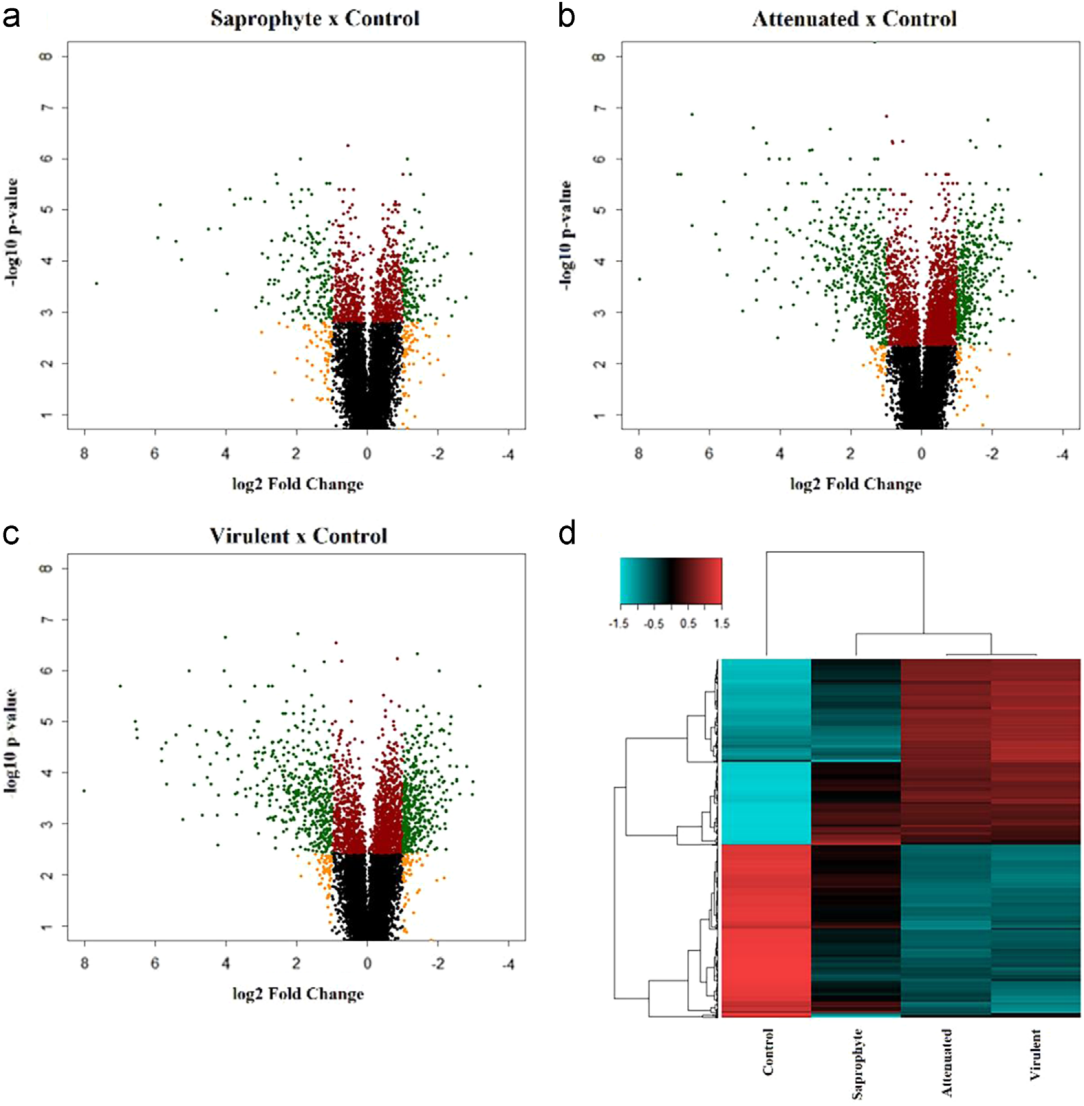
**Distribution of differentially expressed genes. a-c)** Volcano plot of microarray experiment for each treatment (saprophyte, attenuated and virulent) compared to control cells, containing 34.472 probes for murine macrophages. Red circles indicate probes with FDR < 0.05, orange circles are log2 fold change > 1 and green circles are the genes statically significant with both parameters, FDR < 0.05 and log2 fold change > 1. **d)** Heatmap of differentially expressed genes modulated by macrophages at 6 h of infection by different strains of *Leptospira* spp shows the average signal of genes. Upregulated genes are plotted in red color and downregulated genes in blue. (*n* = 3/treatment; *p*-value < 0.01; FDR < 0.05; linear fold change ± 2).

Following normalization, CHP files were utilized to identify differentially expressed genes using the software Transcriptome Analysis Console (Affymetrix). Infection by *L. interrogans* (virulent and attenuated bacteria) caused statistically significant changes in gene expression in more than 800 genes, when compared to non-infected control cells (Fig. 3a–b). Saprophyte *L. biflexa* modulated 200 genes when compared to control (Fig. 3c). Hierarchical clustering and differences in average signal between samples are depicted in a heatmap showing a clear separation of groups based on infection and leptospiral virulence (Fig. 3d). Canonical pathways of the differentially expressed genes were identified using the Ingenuity Pathway Analyses (IPA®) software.

### 2.7 Validation of transcriptome results by qRT-PCR

For validation of microarray data between treatments, M-MLV reverse transcriptase (Thermo Fisher Scientific) and QuantiTect SYBR® Green (Qiagen) were used for preparation of cDNA and qPCR, respectively, according to manufacturer´s instructions. PCR was performed in an MxPRO3005 real-time PCR machine (Agilent, Santa Clara, CA, USA). Standard curves were used for all genes at each individual run, and the expression of candidate genes was evaluated as a ratio to the housekeeping control, which was chosen on previous comparative analysis on these samples, between three housekeeping genes. All primers were designed to span at least one intron, to avoid repeat regions and similarities to other non-specific genomic regions. Murine sequence, available on the University of California, Santa Cruz (UCSC) Genome Browser, was employed for primer design using the Primer3 program [2].

### 2.8 Statistical analysis

Differential expression of each gene was determined by ANOVA with two criteria, a fold change of ± 2 comparing all infected groups to the non-infected control and filtered by FDR-corrected *p*-value < 0.05 Real time PCR data was analyzed using least-squares analysis of variance and the general linear model procedures of SAS (SAS Institute, Cary, NC, USA; *p* < 0.01). Comparison of means was done using Duncan's multiple range test, and significance was set at *p* < 0.05.
