# Supplementary material for: Transcriptome datasets of macrophages infected with different strains of Leptospira spp
Source: Data Brief. 2017 Dec 21;16:1044–50. doi: 10.1016/j.dib.2017.12.042 (PMC5758930; doi:10.1016/j.dib.2017.12.042)
Supplement: Supplementary file 1 — Transparency document [file mmc1.pdf]

The authors, hereby represented by the corresponding author Dra. Flavia Lombardi Lopes, certify that they have NO affiliations with or involvement in any organization or entity with any financial interest), or non-financial interest in the subject matter or materials discussed in this manuscript.

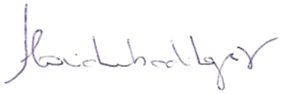

Flavia Lombardi Lopes, DVM, PhD

Departamento de Apoio, Produção e Saúde Animal - FMVA/UNESP

Rua Clóvis Pestana, 793

CEP - 16050-680   Araçatuba - SP

Telefone: 55 (18) 3636-0032

Email: flavialopes@fmva.unesp.br
